# Rapid Engineering of Foot-and-Mouth Disease Vaccine and Challenge Viruses

**DOI:** 10.1128/JVI.00155-17

**Published:** 2017-07-27

**Authors:** Seo-Yong Lee, Yeo-Joo Lee, Rae-Hyung Kim, Jeong-Nam Park, Min-Eun Park, Mi-Kyeong Ko, Joo-Hyung Choi, Jia-Qi Chu, Kwang-Nyeong Lee, Su-Mi Kim, Dongseob Tark, Hyang-Sim Lee, Young-Joon Ko, Min-Goo Seo, Jung-Won Park, Byounghan Kim, Myoung-Heon Lee, Jong-Soo Lee, Jong-Hyeon Park

**Affiliations:** a Animal and Plant Quarantine Agency, Gimcheon City, Gyeongsangbuk-do, Republic of Korea; b College of Veterinary Medicine (BK21 Plus Program), Chungnam National University, Gungdong, Daejon City, Republic of Korea; c Clinical Research Center of the Affiliated Hospital of Guangdong Medical College, Zhanjiang, China; d Korea Zoonosis Research Institute, Chonbuk National University, Iksan Si, Jeollabuk-do, Republic of Korea; University of Southern California

**Keywords:** foot-and-mouth disease, pathogenesis, serotype, synthetic vaccine

## Abstract

There are seven antigenically distinct serotypes of foot-and-mouth disease virus (FMDV), each of which has intratypic variants. In the present study, we have developed methods to efficiently generate promising vaccines against seven serotypes or subtypes. The capsid-encoding gene (P1) of the vaccine strain O1/Manisa/Turkey/69 was replaced with the amplified or synthetic genes from the O, A, Asia1, C, SAT1, SAT2, and SAT3 serotypes. Viruses of the seven serotype were rescued successfully. Each chimeric FMDV with a replacement of P1 showed serotype-specific antigenicity and varied in terms of pathogenesis in pigs and mice. Vaccination of pigs with an experimental trivalent vaccine containing the inactivated recombinants based on the main serotypes O, A, and Asia1 effectively protected them from virus challenge. This technology could be a potential strategy for a customized vaccine with challenge tools to protect against epizootic disease caused by specific serotypes or subtypes of FMDV.

**IMPORTANCE** Foot-and-mouth disease (FMD) virus (FMDV) causes significant economic losses. For vaccine preparation, the selection of vaccine strains was complicated by high antigenic variation. In the present study, we suggested an effective strategy to rapidly prepare and evaluate mass-produced customized vaccines against epidemic strains. The P1 gene encoding the structural proteins of the well-known vaccine virus was replaced by the synthetic or amplified genes of viruses of seven representative serotypes. These chimeric viruses generally replicated readily in cell culture and had a particle size similar to that of the original vaccine strain. Their antigenicity mirrored that of the original serotype from which their P1 gene was derived. Animal infection experiments revealed that the recombinants varied in terms of pathogenicity. This strategy will be a useful tool for rapidly generating customized FMD vaccines or challenge viruses for all serotypes, especially for FMD-free countries, which have prohibited the import of FMDVs.

## INTRODUCTION

The single-stranded positive-sense RNA viruses that cause foot-and-mouth disease (FMD) belong to the Aphthovirus genus of the Picornaviridae family. There are seven serotypes of FMD virus (FMDV), O, A, Asia1, C, SAT1, SAT2, and SAT3, which have been categorized into topotypes (1). FMD is highly contagious and infects cloven-hoofed animals, including cattle, pigs, and goats along with wild animals such as deer. This disease is distributed worldwide, and outbreaks can cause severe country-wide economic damage (2). FMD outbreaks are controlled by the restriction of the movement of susceptible animals, slaughter of infected animals and in-contact animals, and decontamination. Although infected animals are immediately culled during FMD outbreaks, vaccines are needed to suppress the diffusion of FMD in cases where quarantine measures may be delayed. Vaccinations could be an effective method of control, as they can contribute strongly to the prevention and suppression of FMD epidemics (3). The current FMD vaccine, which contains an inactivated whole-virus preparation with an oil adjuvant, is commonly used in enzootic areas. Although the current vaccine has been successfully used to reduce FMD outbreaks, there are a number of disadvantages, including the requirement for an expensive manufacturing facility and differentiating infected from vaccinated animals (DIVA) (4, 5). In addition, because of the emergence of new FMD viruses, vaccination with one serotype cannot confer protection against strains of different serotypes or heterologous strains of the same serotype based on antigenic variation and also cannot respond promptly (5). Therefore, diverse approaches for safe and effective FMD vaccine development, including subunit, recombinant viral, DNA, peptide, and attenuated vaccines, have been extensively studied (6). FMDV particles consist of structural proteins (VP1, VP2, VP3, and VP4) and nonstructural proteins (NSPs) that serve important roles in virus replication. The structural proteins are the main FMDV antigens that are targeted by vaccines (6). If the components of structural proteins could be replaced by a well-characterized strain to fit various serotypes or topotypes, vaccine strains for all types of viruses could be produced effectively within a short period of time. Recently, several studies have used reverse genetics to generate chimeric FMDVs that reproduce the O, A, Asia1, and SAT serotypes (7–12). However, although this technology would assist in creating an effective FMD vaccine that could be applied to new strains, the usefulness of a vaccine strain made from viruses of all serotypes still remains unclear, including the rapid generation of live chimeric viruses and understanding pathological features and serotype-specific immunity through animal experiments.

In the present study, the P1 gene (which encodes VP1, VP2, VP3, and VP4) in an infectious full-length cDNA clone derived from the standard vaccine strain with low-pathogenicity features, O1/Manisa/Turkey/69 (O Manisa) (13, 14), was replaced by the P1 genes of representative viruses of the seven serotypes. The growth characteristics, particle size, and antigenicity of the resulting seven chimeric viruses were assessed. Moreover, the pathogenicity of the recombinants in target and laboratory animal models was investigated. The efficacy of a trivalent vaccine based on three of the inactivated recombinants was also tested in pigs. Collectively, our results provide an innovative vaccine platform that can efficiently and rapidly generate protective vaccines against seven FMDV serotypes or subtypes through the easy exchange of a capsid-encoding gene according to genetic information. Our chimeric viruses can elicit high titers of neutralizing antibodies and confer complete protection against challenge in pigs.

## RESULTS

### Characterization of the chimeric viruses.

**FIG 1:**
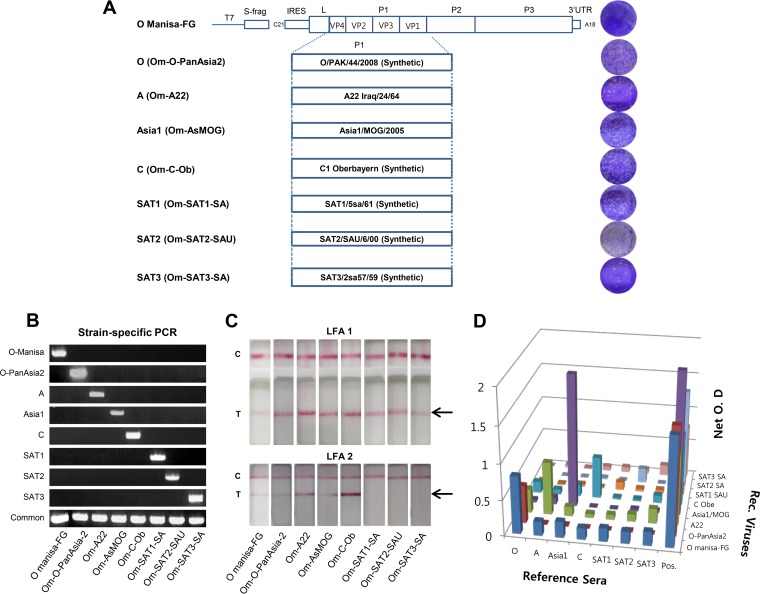
Strategy for obtaining chimeric FMD viruses of seven serotypes and confirming all serotypes. (A) Schematic depiction of the chimeric viruses and their plaques. LF-BK cells were used for the plaque-forming assay for all recombinants except Om-SAT2-SAU, which did not form plaques in these cells. ZZR cells were used to grow the latter recombinant instead. UTR, untranslated region. (B) Genetic differentiation of the VP1 regions by individual-strain-specific RT-PCR. (C) Confirmation of the presence of the appropriate FMDV structural protein in the recombinants by using rapid diagnosis kits (lateral-flow assay) for seven serotypes. In LFA1, the lateral-flow assay was designed to detect all serotypes (Svanova Co. Ltd.). In LFA2, the lateral-flow assay was designed to detect four serotypes (PBM Co. Ltd.). C and T indicate the control and test lines, respectively (structural proteins). (D) Antigen ELISA of the recombinants using reference serotype antisera against O Manisa (O1 BFS 1860 for antigen), A 464, Asia1 CAM 9/80, C3 Resende, SAT1-BOT 1/68, SAT2-ZIM 5/81, and SAT3-ZIM 4/81. O.D, optical density.

**FIG 2:**
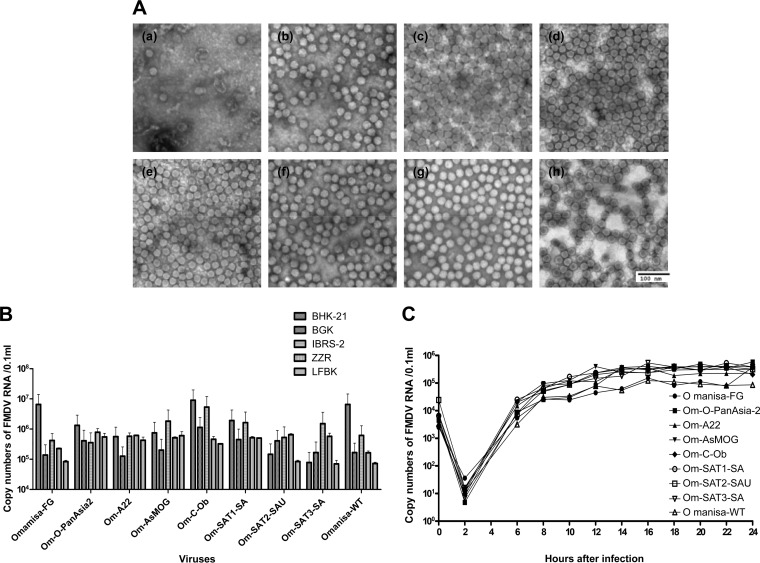
Characteristics of the chimeric FMD viruses produced by replacing the capsid-encoding gene in the O Manisa vaccine strain. (A) Electron microscopy of the viral particles of the recombinants. (a) O Manisa-FG; (b) Om-O-PanAsia2; (c) Om-A22; (d) Om-AsMOG; (e) Om-C-Ob; (f) Om-SAT1-SA; (g) Om-SAT2-SAU; (h) Om-SAT3-SA. (B) Growth properties of the recombinants in BHK-21, BGK, IB-RS-2, ZZ-R, and LF-BK cells after being passaged five times in ZZ-R cells. The viral titers at 36 h postinfection were determined by real-time RT-PCR. (C) One-step growth curves of the recombinants over time in ZZ-R cells. The viral titers were determined by real-time RT-PCR. WT, wild type.

The O Manisa virus is a widely used type O vaccine strain and served as the recombinant backbone in the this study. We successfully constructed P1 recombinant clones, denoted pOm-O-PanAsia2, pOm-A22, pOm-AsMOG, pOm-C-Ob, pOm-SAT1-SA, pOm-SAT2-SAU, and pOm-SAT3-SA (Fig. 1A and B), and all seven FMDV recombinant clones were rescued in BHK T7-9 cells (Fig. 1A). The inserted P1 genes of individual serotypes were proven again by PCR (Fig. 1B) and sequencing of the defined viruses. O Manisa formed relatively small viral plaques, whereas the seven recombinants formed relatively large plaques (Fig. 1A). Next, antigen enzyme-linked immunosorbent assays (ELISAs) and lateral-flow assays (LFAs) were conducted by using reference antigens and antibodies. The chimeric viruses expressed the expected serotype-specific antigens (Fig. 1C and D). Additionally, serological reactivity also showed that the inserted antigens reacted with the characteristic serotypes of individual antigens by cross-neutralization antibody tests (Table 1). In particular, the A22, C, and SAT1 reference sera cross-reacted weakly (virus neutralization [VN] titers of 16 to 64) with the O Manisa chimeric virus. In contrast, the anti-SATI-BOT reference serum reacted most strongly with the SAT1 chimeric virus and showed weak (VN titers of 16 to 64) but broad cross-reactivity with the other chimeric viruses of the O, A, and C serotypes (Table 1). Their virus replication characteristics differed as all the constituting phenotypes were replaced. In addition, electron microscopy revealed that the seven serotypes of viruses differed in size from 23.9 to 29.1 nm (Fig. 2A). When the chimeric viruses were cultured in cell lines derived from FMDV-susceptible species, the Asia1 and SAT3 serotype viruses replicated better in pig-derived IB-RS-2 cells than in hamster-derived BHK-21 cells. However, the recovered viruses that had the phenotypic characteristics of each serotype had similar titers in the five different cell lines (Fig. 2B). All chimeric viruses replicated in ZZ-R cells and induced cytopathic effects (CPEs) (Fig. 2B). In contrast, the SAT2 serotype virus did not induce CPEs in LF-BK cells (Fig. 1A), even though it replicated in this cell line (Fig. 2B). Moreover, the SAT2 and SAT3 serotype viruses failed to replicate in BHK-21 cells for at least three passages.

**TABLE 1:** Serological relationships among FMDV recombinants determined by cross-virus neutralization using reference antisera

Strain against which reference antiserum was raised^*a*^	Reciprocal arithmetic titer for virus used in cross-VNT^*b*^							
	O Manisa-FG	Om-O-PanAsia2	Om-A22	Om-AsMOG	Om-C-Ob	Om-SAT1-SA	Om-SAT2-SAU	Om-SAT3-SA
O1 Manisa	724	362	—	—	—	—	—	—
A22	16	—	90	—	—	—	—	—
Asia Shamir	—	—	—	90	—	—	—	—
C Resende	16	—	—	—	181	—	—	16
SAT1-BOT 1/68	64	22	—	22	16	362	—	—
SAT2-ZIM 5/81	—	—	—	—	—	—	1,448	—
SAT3-ZIM 9/80	—	—	—	—	—	—	—	362

a The reference serum for type O was produced from animals immunized with O1 Manisa, and the reference sera for types A22, Asia1, C, SAT1, SAT2, and SAT3 were obtained from an antigen detection kit.

b VNT, virus neutralization test. —, reciprocal titer of <16 (negative cutoff value for the virus neutralization test).

These results suggest that the seven constructed P1-replaced chimeric viruses are similar to seven wild-type FMDVs in antigenicity.

### Pathogenicity of the chimeric viruses in animals.

**FIG 3:**
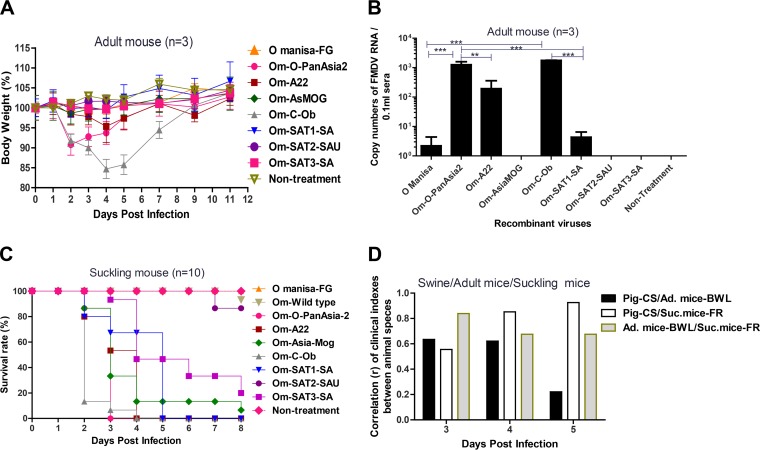
Comparison of pathogenesis in suckling mice, adult mice, and pigs after infection with chimeric FMDVs. Suckling mice (*n* = 10) and adult mice (*n* = 3) were challenged with the recombinant FMDV (10^5.0^ TCID_50_/0.1 ml) intraperitoneally. Pigs (*n* = 2) were challenged with the virus (10^5.0^ TCID_50_/0.1 ml) intradermally in each footpad. (A) Change in adult mouse body weight (percentage) over time after chimeric FMDV challenge. (B) Viremia on the third day after chimeric FMDV challenge in adult mice. *P* values of <0.01 (**) and <0.001 (***) were regarded as significant or highly significant. (C) Survival rate of suckling mice after challenge with recombinant FMDVs and the O Manisa wild type (Om-Wild type). (D) Correlation (*r*) of the relationships between porcine clinical score (CS), body weight loss (BWL) (percent) of adult mice, and fatality rate (FR) (percent) of suckling mice on days 3, 4, and 5 after challenge with eight tested viruses of all serotypes.

To investigate the pathogenicities of the chimeric viruses (O Manisa-FG, Om-O-PanAsia2, Om-A22, Om-AsMOG, Om-C-Ob, Om-SAT1, Om-SAT2, and Om-SAT3), we examined clinical indexes in adult mice, suckling mice, and pigs inoculated with each chimeric FMD virus. The effect of each chimeric virus on the weight of inoculated adult mice (C57BL/6) was then assessed (Fig. 3A). Om-C-Ob induced the most severe weight loss: the mice had lost about 15% of their body weight 4 to 5 days after inoculation. Om-O-PanAsia2 also had a relatively severe effect of a 10% body weight loss (BWL) on the second day. Om-A22 induced slight body weight losses of 5% on the fourth day. The other chimeric viruses did not have a marked effect on weight (Fig. 3A). Assessment of viremia on the third day after inoculation of adult mice revealed that Om-C-Ob and Om-O-PanAsia2 yielded the highest-level viremias, followed by Om-A22 (Fig. 3B). All suckling mice inoculated with Om-C-Ob and Om-O-PanAsia2 died within 2 to 4 days. The mice inoculated with Om-A22, Om-AsMOG, Om-SAT1-SA, and Om-SAT3-SA also died within 2 to 8 days. In contrast, the Om-SAT2 and O Manisa viruses had low pathogenicity (Fig. 3C). Porcine clinical scores (CSs) and suckling mouse fatality rates (FRs) on the fifth day correlated very closely (*r* = 0.925). These data indicate that pig clinical scores and fatality rates for suckling mice had the highest correlation on the fifth day (Fig. 3D).

**FIG 4:**
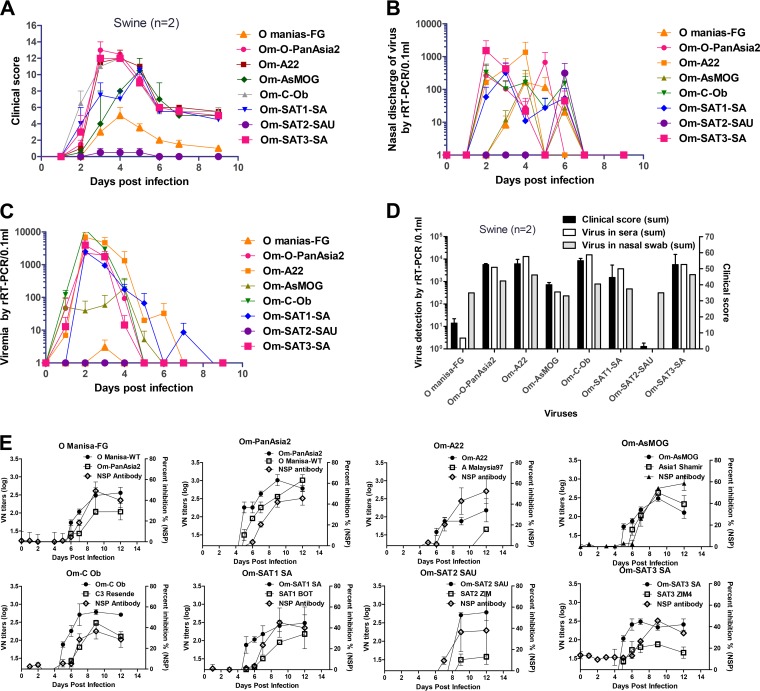
Pathogenesis in pigs infected with chimeric FMDVs. Pigs (*n* = 2) were challenged with the virus (10^5.0^ TCID_50_/0.1 ml) intradermally in each footpad. (A) Clinical scores of chimeric FMDV-challenged pigs over time. All pigs in the experiment were challenged with recombinant FMDV (10^5.0^ TCID_50_/0.1 ml) in each footpad. (B) Detection of virus in nasal discharge by real-time RT-PCR (rRT-PCR). (C) Detection of virus in sera by real-time RT-PCR. (D) Comparison of clinical scores and detection of virus in sera and nasal swabs. Shown are total clinical scores throughout the experimental period, total viremia throughout the experimental period, and total virus counts in nasal discharge during the experimental period in inoculated pigs. (E) Antibody responses (virus-neutralizing titers [left axis] and percent inhibition ratios in an NSP ELISA [right axis]) to the original wild-type serotypes and chimeric FMDV in pigs after chimeric FMDV challenge. The cutoff for a positive reaction in the NSP ELISA is >50% inhibition.

Next, chimeric viruses with various pathogenicities, produced based on low-pathogenicity O Manisa, were generated in the pigs. Clinical symptoms of FMD, viremia, and excretion of viruses through nasal secretions were determined during the experimental period (Fig. 4A to D). O Manisa and Om-SAT2-SAU were the least pathogenic viruses. Om-SAT1-SA and Om-AsMOG had moderate pathogenicity, while Om-C-Ob, Om-O-PanAsia2, Om-A22, and Om-SAT3-SA had the strongest pathogenicity. The sum of the clinical indexes during the experimental period correlated moderately with the total level of viremia in sera during the experimental period (*r* = 0.686). The sum of clinical symptoms during the experimental period also correlated moderately with the total amount of viruses in nasal secretions during the experimental period. There was also a moderate correlation between total viremia and total amounts of virus in nasal secretions. In cases where the animals were inoculated with Om-C-Ob, clinical symptoms (within 1 day) were quickly induced, and severe viremia and a large amount of virus excretion also followed (Fig. 4B). Om-O-PanAsia2, Om-A22, Om-SAT1-SA, and Om-SAT3-SA also induced high-level viremia (Fig. 4C and D). We then assessed the correlations between suckling mouse FRs, adult mouse BWLs, and porcine CSs on days 3, 4, and 5 after inoculation (Fig. 3D). Om-C-Ob had the highest pathogenicity, followed by Om-O-PanAsia2 and Om-A22. Om-SAT2-SAU was the least pathogenic of the seven recombinants. The porcine clinical score and rate of weight loss in adult mice on the third day (*r* = 0.635) and fourth day (*r* = 0.621) correlated moderately well. Adult mouse weight loss and suckling mouse fatality rates on the third day correlated closely (*r* = 0.839). Therefore, these results suggest that the seven constructed chimeric viruses induce various levels of pathogenicity based on the characteristics of the P1 gene of each virus.

### Type-specific antibody responses after infection of pigs.

To confirm the immunological reactivity of the chimeric FMD viruses (O Manisa-FG, Om-O-PanAsia2, Om-A22, Om-AsMOG, Om-C-Ob, Om-SAT1, Om-SAT2, and Om-SAT3), the formation of neutralizing antibody against chimeric viruses and wild-type reference FMDVs of different serotypes in inoculated pigs was then investigated (Fig. 4E).

Antibodies against virus structural proteins were detected on days 5 to ∼7 and reached a peak on day 9. Antibodies against virus nonstructural proteins were detected on days 6 to ∼7, and NSP antibody levels were low or delayed in proportion to pathogenicity in pigs (Fig. 4E). Antibodies raised against O Manisa-FG neutralized Om-O-PanAsia2 equally as well as the chimeric virus and vice versa. Similarly, antibodies raised against Om-AsMOG neutralized Asia1 Shamir equally as well as the inoculating virus. In contrast, the antibodies raised against Om-A22 neutralized A Malaysia97 weakly. The antibodies generated against Om-SAT2-SAU also neutralized SAT2-ZIM weakly. Meanwhile, in the case of the O serotype, intraserotypic cross-reactivity between the O Manisa and Om-O-PanAsia2 viruses and low interserotypic cross-reactivity between A and Asia1 were determined. The abilities of the neutralizing antibodies raised in pigs by inoculation with the chimeric viruses to neutralize other serotypes are summarized in Table 2. These results show that the chimeric viruses generally induced highly neutralizing antibodies against the designated serotypes.

**TABLE 2:** Type-specific antibodies in chimeric FMDV-infected pigs determined by cross-virus neutralization

Chimeric FMDV (infected pigs [*n* = 2])	Reciprocal arithmetic titer for virus used in cross-VNT (pig 1/pig 2)^*a*^							
	O Manisa	Om-O-PanAsia2	Om-A22	Om-AsMOG	Om-C-Ob	Om-SAT1-SA	Om-SAT2-SAU	Om-SAT3-SA
O Manisa (223, 233)	256/512	45/181	—/—	—/—	—/—	—/—	—/—	—/—
Om-PanAsia2 (215, 235)	128/256	724/512	—/—	—/16	—/—	—/—	—/—	—/—
Om-A22 (224, 236)	—/90	—/128	256/90	—/—	—/—	—/—	—/—	—/—
Om-AsMOG (200, 222)	16/16	181/—	—/—	181/90	—/—	—/—	—/—	—/—
Om-C-Ob (212, 218)	—/—	—/—	—/—	—/—	512/512	—/—	—/—	—/—
Om-SAT1-SA (219, 220)	—/—	—/—	—/—	—/—	—/—	181/512	—/—	16/—
Om-SAT2-SAU (291, 234)	—/—	—/—	—/—	—/—	—/—	—/—	362/1,024	—/—
Om-SAT3-SA (189, 217)	—/—	—/—	—/—	—/—	—/—	—/—	—/—	362/181

a VNT, virus neutralization test using sera from the pigs 12 days after infection with each chimeric FMDV. —, reciprocal titer of <16 (negative cutoff value for the virus neutralization test).

### Immune responses and protection of immunized target animals.

**FIG 5:**
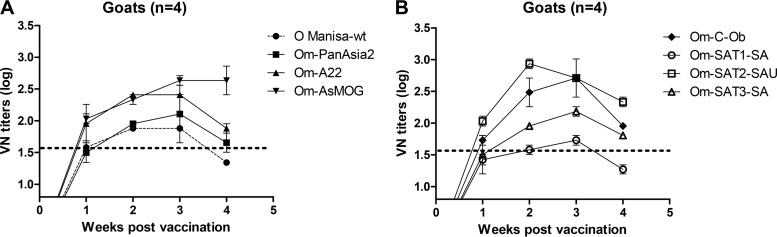
Immune responses after vaccination with chimeric FMDV antigens in dairy goats. Dairy goats (*n* = 4) were inoculated with antigens (each at 5 μg/2 ml) by intramuscular injection. (A) Neutralizing antibody titers against the recombinants of the O, A, and Asia1 types in immunized dairy goats (*n* = 4) that had been immunized once with oil-adjuvanted vaccines based on each of the recombinants. (B) Neutralizing antibody titers against recombinants of the C, SAT1, SAT2, and SAT3 types in dairy goats (*n* = 4).

The chimeric FMD viruses were then inactivated by using a process employed for experimental FMD vaccine production. Also, seven kinds of antigens (except for O Manisa) were purified to remove NSP. Dairy goats (*n* = 4) were immunized once with each preparation together with an oil adjuvant. An analysis 1 week later showed that all recombinant preparations induced protective levels of neutralizing antibodies (VN titer of >45) against the original serotype (Fig. 5). While most preparations induced high neutralizing antibody titers, the SAT1 serotype induced relatively low antibody levels. The recombinants based on the SAT2, C, Asia1, SAT3, A, and O serotypes produced comparatively high neutralizing antibody titers (above 100) within 3 weeks.

**FIG 6:**
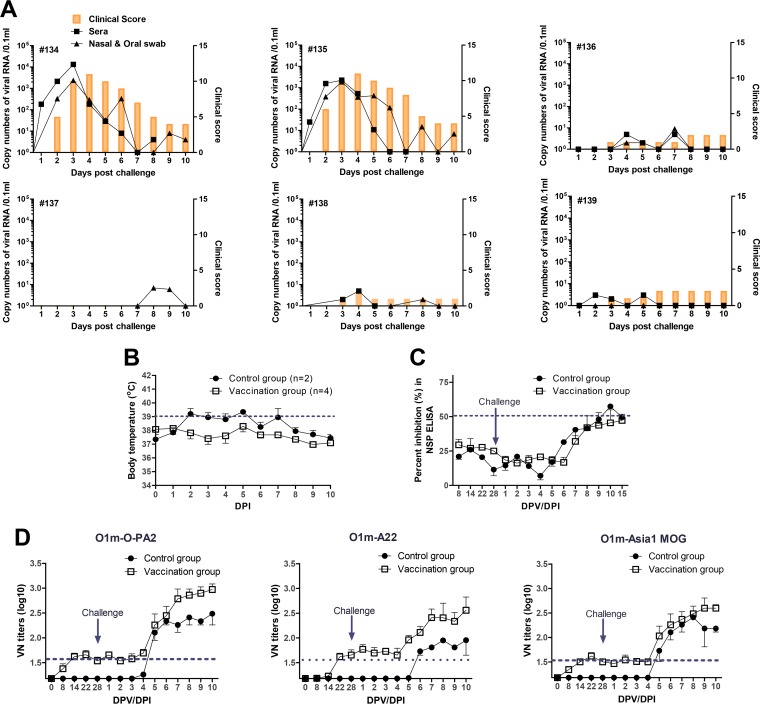
Protection from challenge in pigs vaccinated with an experimental trivalent vaccine based on the Om-O-PanAsia2, Om-A22, and Om-AsMOG recombinants. Pigs (*n* = 4) were vaccinated with the antigen (each 7.5 μg/2 ml) by intramuscular injection. Three chimeric viruses (Om-O-PanAsia2, Om-A22, and Om-AsMOG) were used to challenge pigs intradermally in the footpad with viral titers of 10^5.0^ TCID_50_/0.1 ml on day 28 after vaccination. (A) Pigs 134 and 135 were not vaccinated, while pigs 136 to 139 were vaccinated. The clinical score, amount of virus excreted from the oral cavity, and viremia are shown for each pig. (B) Change in body temperature after challenge in vaccinated and nonvaccinated pigs. The normal range of body temperatures in pigs is 38.7°C to 39.8°C. (C) Titers of antibodies specific for NSPs after vaccination and challenge of vaccinated and nonvaccinated pigs. NSPs were detected by using a PrioCHECK FMDV NSP ELISA kit. (D) VN antibody titers in vaccinated and nonvaccinated pigs after vaccination and challenge. Homologous viruses were used for the test. The cutoff for a positive reaction in the VN test is >1:16, and that for a positive reaction in the NSP ELISA is >50% inhibition. DPV, days postvaccination; DPI, days postinfection.

We next evaluated the protective efficacy of an experimental trivalent vaccine that contains the three main antigens derived from the chimeric FMDVs (Om-PanAsia2, Om-A22, and Om-AsMOG) of the O, A, Asia1 serotypes, which frequently occur in Asian countries. Pigs were immunized with the trivalent vaccine and then challenged with a mixture of these three viruses (Fig. 6). Apart from lesions at the injection sites, the vaccinated animals (animals 136 to 139) did not develop any severe clinical signs of FMD. In contrast, the nonvaccinated animals (animals 134 and 135) showed typical clinical signs of FMD starting on day 2 postinfection, and high levels of virus were detected in their sera and oral cavities (Fig. 6A). The nonvaccinated animals also exhibited an elevated body temperature starting on day 2 postinfection. In contrast, the vaccinated group did not show any changes in body temperature during 10 days after challenge (Fig. 6B). With little difference among the three chimeric viruses, the viruses of the O, A, and Asia1 serotypes induced similar titers of neutralizing antibodies 2 to 3 weeks after vaccination. The neutralizing antibody levels of the vaccinated group were higher than those of the nonvaccinated group. After challenge, the neutralizing and NSP antibody levels of both groups increased at 5 and 6 to ∼7 days postinfection (dpi), respectively (Fig. 6C and D). Taken together, these result indicate that immunization with an experimental trivalent vaccine induced a potential immune response that conferred the expected level of antibody (VN titer of >45) for protection against FMD viruses.

Collectively, these results demonstrate that the chimeric FMD viruses can induce neutralizing antibodies that are highly specific for the original serotype in pigs and goats.

## DISCUSSION

Previous studies attempted to generate chimeric FMDVs for efficient vaccine development in pigs or cattle. Attenuated, antigenically marked viruses formed by mutation of 3B and 3D sites have already been made for A24 virus strain-based leader sequence deficits and markers (12). In another experiment, the P1 genes of the O-UKG and A-TUR viruses were replaced with the P1 gene of the O1K strain (15). In another study, only the P1 gene of the complete genome of SAT2/ZIM/07/83 was replaced with the P1 antigen of other SAT2 serotype strains or SAT3/ZAM/4/96 (16).

The findings of the present study support the notion that an effective vaccine strain can be generated quickly by replacing the P1 gene of O Manisa with synthetic capsid-encoding regions of all serotypes or topotypes of specific FMDVs according to genetic information. The chimeric clones were expected to replicate as quickly as O Manisa because the chimeric viruses shared the nonstructural proteins of O Manisa, which is well adapted to replication in cell lines originating from goats, hamsters, pigs, and cattle. However, the chimeric viruses showed some differences in terms of replication in different cell lines; for example, some of the viruses replicated much faster in BHK-21 than in IB-RS-2 cells. These observations suggest that the P1 gene is involved in the efficiency of the FMDV replication cycle in cells. Because the chimeric clones had same sequences of nonstructural proteins and untranslated regions, we speculated that these sequences did not affect differences in replication. All chimeric viruses had type-specific characteristics as a vaccine antigen. Since a BHK-21 cell line is used for vaccine production, it is important that the recombinants can grow well these cells. However, while most viruses grew well in BHK-21 cells, the chimeric viruses based on the SAT2 and SAT3 serotypes failed to replicate in BHK-21 cells with at least three passages. This is not surprising because the serotype SAT2 and SAT3 strains used in this experiment were not adapted in cells, and it is well known that it is difficult to adapt SAT-type viruses to cell culture (17–19). Nevertheless, this characteristic should be considered when producing recombinant vaccine strains in the future.

In our pathogenicity experiments, the chimeric viruses differed markedly in terms of pathogenicity. Since the chimeric viruses differed only in terms of their P1 regions, this suggests that the P1 region is correlated closely with FMDV pathogenicity. This result is consistent with previously reported findings for cattle (15). In particular, the SAT2 serotype-based chimeric virus, whose replication characteristics differed from those of the other recombinants, was relatively nonpathogenic. It induced little mortality in suckling mice and did not induce any viremia or clinical symptoms in pigs or adult mice. However, this virus replicated in pigs, as it was excreted in the nasal discharge on the sixth day. Moreover, this chimeric virus induced high neutralizing antibody titers only on the eighth day after inoculation (about 4 days later than for the other recombinants). In contrast, Om-C-Ob and Om-A22 induced high-level viremia in pigs within 2 days, which indicates very fast *in vivo* replication and high pathogenicity, like the virulence of the wild type. Om-O-PanAsia2 was also highly pathogenic. Even though in incomplete accord, these differences may reflect the abilities of these chimeric viruses to replicate in different cell types. These differences probably also reflect differences in the P1 genes in terms of their effects on virus pathogenicity. The latter notion is supported by the fact that VP1 is associated with the immunogenicity of FMDVs and includes the Arg-Gly-Asp (RGD) sequence that reacts with integrin as a cellular receptor (20–23). In pathogenicity experiments, we also observed that pig clinical scores and mortality rates in suckling mice had the highest correlation on the fifth day. These results could be useful for further vaccine studies. Because the animals immunized with live virus had higher VN antibody titers than did those vaccinated with an inactivated antigen, live virus would be more effective for the control of FMD. Since these strains could potentially protect animals as early as 2 days after vaccination, the attenuated virus could control a disease outbreak very quickly (24). Cross-reaction experiments with reference antisera against the serotypes that were used to generate the chimeric viruses revealed that they neutralized the chimeric viruses in a serotype-specific manner: the reference sera demonstrated little cross-reactivity with the other chimeric viruses. This finding suggested that these chimeric viruses are unlikely to provide cross-protection when they are used individually as vaccines. The chimeric viruses generally induced highly neutralizing antibodies against the designated serotypes in pigs. However, Om-A22 and A Malaysia97 or Om-SAT2-SAU and SAT2-ZIM showed a low correlation in neutralizing ability. Dairy goats immunized with each inactivated chimeric viral antigen, including Om-SAT2-SAU, were not used for challenge tests because of inapparent infection. Such characteristics showed that these antigens had traits that made them worthy vaccine candidates, with low pathogenicity and the induction of highly neutralizing antibodies that were highly specific for the SAT2 serotype. Antibodies raised by O Manisa-FG infection in pigs neutralized Om-O-PanAsia2 equally as well as they neutralized the inoculating recombinant and vice versa. Similarly, antibodies raised against Om-AsMOG in pigs neutralized Asia1 Shamir equally as well as they neutralized the inoculating virus. This cross-reactivity pattern was similar to that seen in the reference antiserum: the A22, C, and SAT1 serotype sera cross-reacted weakly with the O Manisa sera. The slight cross-reactivity between the O, A, Asia1, and C serotype sera is probably due to the fact that these viruses are Euro-Asiatic types.

In the present study, customized vaccine strains were generated by using nucleotide sequence information only. Subsequent experiments showed that the chimeric viruses were generated by replacing the P1 gene of the O Manisa vaccine strain with the P1 genes of all seven serotypes without affecting capsid formation or antigenicity. Moreover, virus challenge in pigs vaccinated with an experimental trivalent vaccine (types O, A, and Asia1) showed that this vaccine was protective against three serotypes. In comparisons of the immunities of goats and pigs, VN titers in goats were higher than those in pigs. To induce enough of an immune response, small ruminants are vaccinated with one-half or one-third of cattle dose of oil-based or aqueous vaccines, respectively (25).

These results show that the preparation of backbone virus clones can facilitate the urgent production of vaccine strains. Also, this experimental method can be used to rapidly generate new vaccines by simply removing their leader sequences only or by modifying 3B to remove their pathogenicity, as appropriate. Future research is needed to establish a system that produces FMDVs that yield the same virus titers as those of the parental virus when they are grown in BHK-21 cells, which are used for vaccine production. Moreover, a system that produces nonpathogenic recombinant vaccine strains that bear marker genes is needed. These systems will improve the production of safe and powerful FMD vaccines within at least 6 months.

In conclusion, our results provide an innovative vaccine platform that can efficiently and rapidly generate protective vaccines against seven FMDV serotypes or subtypes just by the easy exchange of the capsid-encoding gene according to genetic information. Our chimeric viruses can elicit high titers of neutralizing antibodies and confer complete protection against challenge in pigs. This strategy may be useful for the development of challenge viruses and a new vaccine to expedite epidemic FMD control.

## MATERIALS AND METHODS

### Viruses and genes.

We inserted the complete genome (GenBank accession no. AY593823) of FMDV serotype O strain O1 Manisa/Turkey/69 (O Manisa) into the cloning vector. This genome is from the most frequently used representative vaccine strain for FMDVs and was cloned into the pBluescript SK II vector (Stratagene) to construct full-length infectious cDNA clones (pO Manisa-FG) of pO Manisa (14). The pO-Manisa-FG P1 genes, which encode the viral structural protein, were replaced by the P1 genes of seven serotypes of FMD viruses (Fig. 1A): O PAK/44/2008 (GenBank accession no. GU384682) for the PanAsia-2 lineage, A22 Iraq/24/64 (GenBank accession no. AY593764), Asia1/MOG/2005 (GenBank accession no. EF614458.1), C1 Oberbayern iso88 (GenBank accession no. AY593805), SAT1/5sa/61 (GenBank accession no. AY593842), SAT2-SAU/6/00 (GenBank accession no. AY297948), and SAT3-2sa57/59 (GenBank accession no. AY593850). The criteria for the selection of each strain were (i) commercialized strains (types O, A, and C) used in international antigen banks, (ii) local strains occurring in Asian countries (type Asia1), and (iii) typical topotypes (strains) frequently occurring in the African continent (topotype III for SAT1, topotype VII for SAT2, and topotype I for SAT3).

### Cloning of virus genes and rescue of chimeric viruses.

The P1 genes of A22 Iraq/24/64 and Asia1/MOG/2005 were secured by PCR using Phusion high-fidelity DNA polymerase (Thermo Fisher Scientific) according to the manufacturer's instructions. Amplification of the capsid-encoding genes of all seven serotypes was performed by using the primers described in Table 3.

**TABLE 3:** Primers used to replace the P1 genes of seven serotypes in pO Mamisa-FG

Serotype or strain	Forward primer	Target region for forward primer	Reverse primer	Target region for reverse primer
O Manisa	5′-CTTCTAAATTTTGACCTGCTCAAAT-3′	2A	5′-CTTGAGCCTTTTCTGGACCTTTGT-3′	L
O-PanAsia2	5′-GGCGCCGGGCAATCCAGCCCGGCG-3′	VP4	5′-CTGTTTCACAGGTGCCACTATCT-3′	VP1
A22	5′-GGAGCCGGGCAATCCAGTCCGGCG-3′	VP4	5′-TTGTTTTGCAGGTGCAATGATCTTC-3′	VP1
AsMOG	5′-GGAGCCGGGCAATCCAGTCCGGCG-3′	VP4	5′-CTGTTTCTCAGGTGCAATGATCTCC-3′	VP1
C-Ob	5′-GGAGCTGGGCAATCCAGCCCAGCG-3′	VP4	5′-TTGTTTTGCAGGTGCGACGAGCGG-3′	VP1
SAT1-SA	5′-GGAGCAGGTCAGTCGTCACCAGCT-3′	VP4	5′-CTGCTTGGCAGGTTTGACGAGGGC-3′	VP1
SAT2-SAU	5′-GGAGCCGGGCAATCCAGCCCAGCC-3′	VP4	5′-CTGCCTCTCGACGCCAATGGGCGC-3′	VP1
SAT3-SA	5′-GGAGCGGGTCAGTCGTCCCCTGCC-3′	VP4	5′-TTGTTTGTCAGGTGCCACCAGTTTG-3′	VP1

The P1 genes of O PAK/44/2008, C1 Oberbayern iso88, SAT1/5sa/61, SAT2-SAU/6/00, and SAT3-2sa57/59 were synthesized by a biotechnology company (Bioneer Co. Ltd.) according to gene sequences in the GenBank database. PCR using the primers (pO Manisa) in Table 3 as insertion vectors was then conducted to amplify pO Manisa-FG clones, except for their P1 genes. The P1 genes of the individual serotypes were then ligated with the vectors by using a TaKaRa Long DNA ligation kit. The cloned plasmids were linearized by treatment with the restriction enzyme SpeI (NEB). BHK T7-9 (baby hamster kidney) cells that stably express T7 RNA polymerase (26) were transfected by using Lipofectamine 2000 (Invitrogen) with these linearized plasmids to recover seven types of chimeric viruses. These chimeric viruses were then passaged five times in the ZZ-R fetal goat tongue cell line (27), and the replication cycle of the generated viruses was determined.

### Identification of replaced P1 genes in the chimeric viruses.

To detect the genes of antigens of individual serotypes, the common 2B gene sequence was amplified by using the reverse primer 5′-AAGGTGCTGTCCAGAATC-3′ and VP1 forward primers specific for individual serotypes (5′-TCACCCGGACCAGGCTAG-3′ for O Manisa, 5′-TCACCCGAGCGAAGCAAG-3′ for O-PanAsia2, 5′-GGAGGTGTCGTCTCAAGAC-3′ for A22, 5′-TGACACCACACAAGACCG-3′ for Asia1, 5′-TCAGCCAACGGGTGATAG-3′ for C, 5′-GCACTACGACCACGGCAACAA-3′ for SAT1, 5′-GCTGCTGCCAGCCTATGAACA-3′ for SAT2, and 5′-ACACGCATACCACTGACC-3′ for SAT3) to confirm the accurate replacement of capsids from passaged chimeric viruses. Conventional RT-PCR was conducted to determine the specific individual serotypes of genes. The universal sequence of the virus was amplified with internal ribosome entry site (IRES) forward primer 5′-GCCTGGTCTTTCCAGGTCT-3′ and reverse primer 5′-CCAGTCCCCTTCTCAGATC-3′ by using One-Step RT-PCR (Qiagen) for a positive control.

### Virus growth and morphology of chimeric viruses.

The ZZ-R (fetal goat tongue), BHK-21 (baby hamster kidney), IB-RS-2 (swine kidney) (33), LF-BK (bovine kidney) (28), and BGK (black goat kidney) cell lines were used to examine the viral growth characteristics of the recovered FMDVs in various cells. Virus titers were quantified by using real-time RT-PCR according to the time course. After the viruses proliferated in cells, plaque assays were conducted with LF-BK or ZZ-R cells in order to determine viral plaque morphologies.

Before the viruses were observed with an electron microscope, BHK-21 or ZZ-R cells were inoculated with the viruses when the cells formed a monolayer in a 175-cm^2^ T flask. When a 100% CPE was observed 24 h later, the viruses were kept in a freezer at −70°C. The viruses were harvested, binary ethyleneimine (BEI) inactivated, concentrated by using polyethylene glycol 6000 (PEG 6000), and then purified by sucrose density gradient centrifugation in an ultracentrifuge (29). The viruses were concentrated using by Amicon ultracentrifuge filters (100 kDa). Samples were observed by using electron microscopes.

### Identification of antigen by ELISAs and lateral-flow assays.

To distinguish the seven serotypes, an antigen ELISA (World Reference Laboratory for FMD [WRLFMD], Pirbright, UK) of the recombinants using reference serotype antisera (rabbit and guinea pig) against O Manisa (O1 BFS 1860 for antigen), A 464, Asia1 CAM 9/80, C3 Resende, SAT1-BOT 1/68, SAT2-ZIM 5/81, and SAT3-ZIM 4/81 was conducted by using methods suggested by the WRLFMD.

Structural proteins were checked by using an FMDV lateral-flow assay (30, 31) in order to determine the expressions of structural proteins of viruses of various serotypes. In LFA1, the lateral-flow assay was designed to detect all serotypes (Svanova Co. Ltd.). In LFA2, the lateral-flow assay was designed to detect four Euro-Asiatic serotypes (O, A, Asia1, and C types) (PBM Co. Ltd.).

### Serotype specificity for the seven serotypes.

We compared the levels of neutralizing antibodies in sera from the animals infected with the chimeric viruses and neutralizing antibodies in standard reference sera of all serotypes (O Manisa, A Malaysia97, Asia1 Shamir, C3 Resende, SAT1-BOT, SAT2-ZIM, and SAT3-ZIM). The neutralizing antibody level for the cross-VN test was determined according to methods in the FMD manual of the World Animal Health Organization (OIE) (34).

### Pathogenesis and clinical signs in animals.

In order to determine the pathogenicities of chimeric viruses of the seven serotypes in animals (pigs [*n* = 2], mice [*n* = 3], and suckling mice [*n* = 10]), two pigs each were inoculated with viruses of each serotype in the footpad, which is a region sensitive to FMDV, at 10^5.0^ 50% tissue culture infective doses (TCID_50_)/0.1 ml and observed for 2 weeks. The clinical score was determined by the addition of points distributed as follows: (i) an elevated body temperature of 40°C (score of 1), >40.5°C (score of 2), or >41°C (score of 3); (ii) reduced appetite (1 point) or no food intake and food left over from the day before (2 points); (iii) lameness (1 point) or reluctance to stand (2 points); (iv) the presence of heat and pain after palpation of the coronary band (1 point) or not standing on the affected foot (2 points); (v) vesicles on the feet, dependent on the number of feet affected, with a maximum of 4 points; and (vi) visible mouth lesions on the tongue (1 point), gums or lips (1 point), or snout (1 point), with a maximum of 3 points (32). After challenge inoculation, levels of virus in nasal discharge and serum samples were monitored for 9 days by collecting the samples at 1-day intervals; viruses were detected by using the real-time RT-PCR method.

After intraperitoneal inoculation of 10^5.0^ TCID_50_/0.1 ml, fatality rates and weight changes in C57BL/6 mice (*n* = 3) were observed. After virus challenge, survival rates and weight changes in adult mice (C57BL/6) were observed until the 12th day after inoculation. Suckling mice (BALB/c) (*n* = 10) sensitive to FMDV were inoculated with 10^5.0^ TCID_50_/0.1 ml at 7 days of age, and only their survival rates were investigated for 8 days. The PrioCHECK FMDV NSP kit (Prionics AG, Schlieren-Zurich, Switzerland), an ELISA kit for the detection of FMDV NSP antibodies in serum samples of pigs and goats, was employed to detect NSP antibodies until the 12th day postinfection, because the antibody could generally be detected at 10 to ∼12 dpi.

### Challenge of pigs immunized with the experimental trivalent vaccine.

The chimeric FMDVs of the O, A, and Asia1 serotypes (Om-PA2, Om-A22, and Om-AsMOG) were cultured in ZZ-R cells and BHK-21 cells, and the virus was inactivated by BEI treatment and incubation at 37°C for 24 h to a final concentration of 0.003 N, according to the same method used to produce trivalent FMD vaccines. The three antigens were refined so that NSP was removed. Pigs were kept under specific-pathogen-free conditions at the institute and used at 3 months of age. Four pigs were then inoculated by intramuscular injection with Emulsigen-D plus 10% aluminum hydroxide (7.5 μg and 2 ml, respectively) (ED+AL), according to the above-mentioned method. In order to determine antibody levels and virus excretion after vaccination or challenge with three homologous viruses (each at 10^5.0^ TCID_50_/0.1 ml) in the footpad, sera were collected for 4 weeks after vaccination (0, 7, 14, 21, and 28 days postvaccination [dpv]), and sera and oral swabs were collected for 10 to 15 days (every day) after challenge, because the antibody could generally be detected by 10 to 12 dpi. Three chimeric viruses (Om-O-PanAsia2, Om-A22, and Om-AsMOG) at 10^5.0^ TCID_50_/0.1 ml were injected intradermally into the footpad for challenge on day 28 after vaccination. The clinical score was determined by the addition of points distributed as described above (32).

### Immunity in dairy goats vaccinated with antigens of the seven serotypes.

The chimeric FMDVs were cultured in ZZ-R cells and BHK-21 cells, and the viruses were inactivated by using the above-mentioned methods. According to the same method as the one used to produce FMD vaccines, the seven antigens (except for O Manisa) were purified so that NSP was removed. Dairy goats were kept under specific-pathogen-free conditions at the institute. Four dairy goats were then inoculated with ED+AL, a mixture of 20% Emulsigen-D (MVP Technologies), which is an oil adjuvant, and 10% aluminum hydroxide [Al(OH)_3_] (Rehydragel HPA; General Chemical) (each at 5 μg/2 ml) by intramuscular injection. Generally, because dairy goats have been shown not to have clinical signs of FMDV infection, we did not conduct virus challenge after vaccination. In order to determine neutralizing antibody titers after inoculation, sera were collected for 4 weeks at 1-week intervals, and sera were kept in a freezer at −70°C. Seven chimeric viruses (Om-O-PanAsia2, Om-A22, Om-AsMOG, Om-C-Ob, Om-SAT1-SA, Om-SAT2-SAU, and Om-SAT3-SA) and O Manisa were used for the test.

### Ethics statement.

Animal experiments were performed in strict accordance with recommendations of the guide for the care and use of laboratory animals of the Animal and Plant Quarantine Agency (QIA). All animal procedures were approved by the Institutional Animal Care and Use Committee of the QIA of South Korea (approval no. 2015-02). All efforts were made to minimize animal suffering.

### Statistical analyses.

Data are presented as means ± standard deviations (SD) and represent results from at least 3 independent experiments. Differences between groups were analyzed by analysis of variance (ANOVA), and means were compared by Student's *t* test. *P* values of <0.05, <0.01, or < 0.001 were regarded as significant or highly significant.
